# Human health-risk assessment of heavy metal–contaminated soil based on Monte Carlo simulation

**DOI:** 10.1038/s41598-023-33986-3

**Published:** 2023-04-29

**Authors:** Ye Panqing, Abdugheni Abliz, Sun Xiaoli, Halidan Aisaiduli

**Affiliations:** 1 College of Geography and Remote Sensing Science, Xinjiang University, Urumqi, 830046 China; 2 Ecological Post-Doctoral Research Station, Xinjiang University, Urumqi, 830046 China

**Keywords:** Environmental chemistry, Environmental impact, Solid Earth sciences

## Abstract

Soil contamination soils of by heavy metals (HMs) poses serious threats to the soil environment and enters the human body through exposure pathways such as ingestion and skin contact, posing a threat to human health. The purpose of this study was to analyze the sources and contributions of soil HMs, and to quantitatively assess the human health risks of soil HMs to different populations (i.e. children, adult females and adult males), and to analyze the human health risks caused by various sources of sensitive populations. 170 topsoil (0–20 cm) were collected from Fukang, Jimsar and Qitai on the northern slope of Tianshan Mountains in Xinjiang, China, and the contents of Zn, Cu, Cr, Pb and Hg were determined. This study used the Unmix model and a health-risk assessment (HRA) model to assess the human health risks of five HMs. The results showed that: (1) The mean values of Zn and Cr were lower than the background values of Xinjiang, the mean values of Cu and Pb were slightly higher than the background values of Xinjiang but lower than the national standard, and the mean value of Hg and Pb was higher than the background value of Xinjiang and the national standard. (2) The sources of soil HMs in the region were mainly traffic, natural, coal, and industrial sources. Moreover, the HRA model combined with Monte Carlo simulation showed similar trends in the health-risk status of all population groups in the region. Probabilistic HRA revealed that noncarcinogenic risks were acceptable for all populations (HI < 1) while carcinogenic risks were high (children: 77.52%; female: 69.09%; male: 65.63%). For children, carcinogenic risk from industrial and coal sources exceeded the acceptable threshold by 2.35 and 1.20 times, respectively, and Cr was the main element contributing to human carcinogenic risk. These findings suggest that carcinogenic risks from coal-based Cr emissions cannot be ignored, and the study area should aim to control Cr emissions from industrial sources. The results of this study provide support for the prevention of human health risks and the control of soil HMs pollution across different age groups.

## Introduction

Soil plays an important role in the material cycles of the terrestrial ecosystem^1^. However, with rapid urbanization and the overexploitation of mineral resources, many heavy metals (HMs) enter the soil^2,3^. HMs in soil do not easily degrade, and they accumulate in the food chain, potentially causing serious harm to human health^4^. Many studies have shown that HMs beyond the threshold can harm humans. For example, excessive amounts of Cr, Cu, and Cd in the environment pose certain noncarcinogenic damage, and liver disease^5^. Meanwhile, HMs such as Pb and Cd are potential carcinogens. Cd entering the human body can lead to lung cancer, hypertension, and renal dysfunction. Pb can lead to neurological, kidney, and gastrointestinal diseases^6,7^. While Zn is an essential element of the human body, excessive intake of Zn can lead to loss of appetite, diarrhea, and anemia, and Hg can cause Minamata disease. Thus, the problem of soil HM pollution has received extensive attention worldwide^8^. HMs in the soil can enter the human body through ingestion, dermal contact, and inhalation. Therefore, to control pollution, it is important to quantitatively identify the sources of HMs in soils, assess the potential health risks of each source, and identify priority control factors^9^.

Source apportionment methods for HMs include qualitative and quantitative pollution source identification^10,11^. Qualitative methods mainly identify possible pollution sources and heavily HM-polluted areas using geostatistics^12^. However, such methods have difficulty analyzing the contribution rate of each pollution source to provide scientific guidance for pollution control. The receptor model, meanwhile, can give the contribution rate of possible sources based on the concentration of multiple pollutants. Common receptor models include APCS-MLR, PMF, Unmix, and CMB^13–15^. Unmix is a receptor model recommended by the US Environmental Protection Agency (USEPA) for quantitatively analyzing pollutant sources. It was first used to quantitatively identify air pollutant sources. In recent years, some studies have applied it to the source analysis of soil HM pollution^16^. Most previous health-risk assessment (HRA) methods for HMs were concentration-driven^17^. The sources of HMs in soil can be divided into both natural and artificial sources. Since it is difficult to intervene in natural sources, the control of HMs in soil has mainly focused on artificial sources^18^. It is difficult, however, to determine the contribution of each source in concentration-oriented HRA, which poses difficulties for decision-making regarding reducing HM risks in the soil. In addition, the HRA of HMs should adopt appropriate strategies. The traditional health risk assessment models mainly rely on fixed exposure parameters and pollutant concentrations, and assume that the exposure parameters are the same for all children, adult males, and adult females. This approach may lead to inaccurate assessment results. However, uncertainty analysis models, such as Monte Carlo risk simulation is an effective way to address such problems^19^. Compared with other uncertainty analysis models, the Monte Carlo method can achieve more accurate analysis results with fewer sample data. In recent years, it has been widely used in the HRA of HMs in soil and water^20–22^. However, there have been few studies on the human health risks of HMs in soil using Monte Carlo simulation combined with source analysis. The contribution rate of heavy metal pollution sources to regional human health risks is still unclear. Monte Carlo simulation combined with source analysis can explain the contribution rate of heavy metal sources to soil pollution and the resulting pollution to human health risks. It can also eliminate to a certain extent the problem of inaccurate human health risk assessment caused by fixed parameters^23^.

The economic belt of the northern slope of the Tianshan Mountains is located in Xinjiang Uygur Autonomous Region, China. This region is far from the sea and has scarce precipitation. It has a typical temperate continental climate and a fragile ecological environment. This economic belt is the most developed area in Xinjiang, with the densest population and the most extensive industrial and agricultural activity. The area accounts for 69.1% of Xinjiang’s GDP. The GDP of the core Urumqi–Changji area accounts for 37.6% of the region’s GDP, and its population accounts for 38.9% of that of Xinjiang. Meanwhile, large amounts of HMs are discharged into the soil in this area. This study aimed to accurately assess soil HM–related health risks for people living in this area. It provides scientific guidance for decision-makers in the region to govern the area and reduce human health risks of soil HMs through scientific means. This study aims to (1) identify and quantify pollution sources by using the Unmix model for source apportionment of soil HMs; (2) assess the human health risks of soil HMs for different population groups in the study area using Monte Carlo simulation, and evaluate the health risk status of each population group; and (3) taking the sensitive population in the study area as an example, use Monte Carlo simulation to evaluate the human health risks of various sources of heavy metals and determine the primary pollution source for control.

## Materials and methods

### Study area

**Figure 1:**
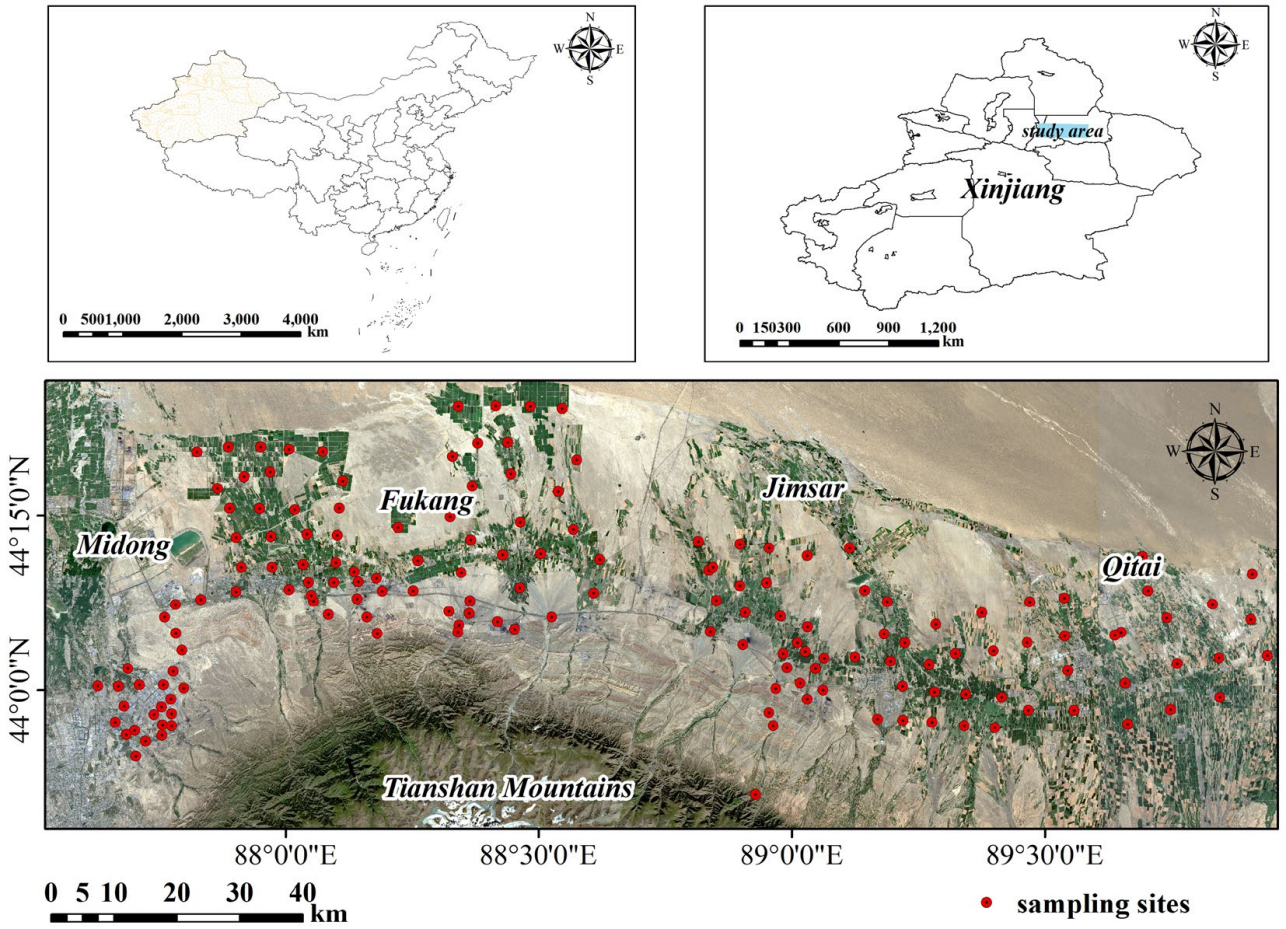
Sketch map of the study area. Map generated with ArcGIS 10.7 (ESRI).URL: https://support.esri.com/en/Products/Desktop/arcgis-desktop/arcmap.

Fukang, Jimsar, Qitai, and Midong are located in the middle of the northern slope of the Tianshan Mountains and the southern margin of the Junggar Basin desert (Fig. 1). Urbanization in this area is rapid and much higher than the average level in Xinjiang^24^, and productivity in the area is highly concentrated. It is the leading area for the development of modern industry, agriculture, and transportation in Xinjiang. It also has the largest integrated coal field in China—the Zhundong coal field—which is an important energy base for power and gas transmission^25^. Given the high intensity of human activity, the soil in this area is seriously polluted by HMs, and the human health risk is high.

### Measurement methods for HM concentration

Based on a comprehensive survey of the study area, sampling was conducted in July 2019, with the aim of distributing the sampling points as evenly as possible. The sampling quadrats were set according to the diagonal method, and the size of the quadrats was determined to be 10 m × 10 m. Five samples were collected from each quadrat according to the diagonal line; only topsoil (0–20 cm) was taken during collection. Five samples in the quadrat were mixed evenly as a representative sample of the sampling point and stored in a clean self-sealing bag. Each representative sample included about 1 kg of original soil samples. GPS was used to locate and record the latitude and longitude of the sampling sites. A total of 171 samples were collected from the study area. Figure 1 shows the distribution of sampling points in the study area. The collected soil samples were pretreated after natural air drying in a dark and ventilated laboratory. First, impurities such as stones, plant impurities, and plastic fragments in the soil samples were sieved. The soil was then ground and passed through a 100-mesh sieve. To avoid introducing other impurities during the grinding process, corundum mortar was used for grinding. Finally, about 100 g of soil was used to determine HM concentration. After digestion with HNO_3_, four HMs (Zn, Cu, Cr, and Pb) were determined by flame atomic absorption spectrometry (Hitachi Z-2000 atomic absorption spectrophotometer, Tokyo Hitachi High-Tech Co., Ltd.). Hg was detected by atomic fluorescence spectrometry (Hitachi Z-2000 atomic absorption spectrophotometer). For the whole analysis process, the national soil primary standard material (GSS-1) was used as the quality control standard. The recovery rate of all elements was within the range of 100% ± 10%.

### Research methods

#### Unmix model

Unmix identifies pollution sources and their contributions using self-modeling curve analysis^26^. The model assumes the data are a linear combination of an unknown number of mixed sources, and the contribution of different sources to each sample is unknown^27^. The principle can be expressed by the following formula:

1 $$C_{ij} = \sum\limits_{k = 1}^{m} {G_{ik} p_{kj} + e}$$

where *C*_*ij*_ is the concentration of* j* in sample *i*, *G*_*ik*_ is the contribution of source *k* in *i* samples, *p*_*kj*_ is the mass fraction of item *j* in source *k* (i.e., the composition of the source), and *e* is the error of model estimation.

#### HRA model

Human health risks include carcinogenic risks (CR) and noncarcinogenic risks (NCR), which are generally calculated by the HRA model provided by the USEPA^28^. CR assesses the probability that an individual will develop cancer owing to long-term exposure to a specific pollutant or mixture of pollutants. NCR is related to individual chronic exposure, including genetic and teratogenic effects. To assess the health risks posed by HMs in soil, the population was divided into three groups: females, males, and children. Different from atmospheric particulates, soil HMs have little risk of inhalation exposure. Therefore, the average daily exposure dose (*ADD*) considers only two main exposure pathways: ingestion and dermal contact^29^. The calculation formula is as follows^30^:

2 $$ADD_{ing} \, = \, \frac{{C_{soil} \, \times \, IR_{ing} \, \times \, CF \times \, EF \, \times \, ED \, }}{BW \, \times \, AT}$$

3 $$ADD_{dermal} \, = \, \frac{{C_{soil} \, \times \, CF \times \, SA \, \times \, AF \, \times \, ABF \, \times \, EF \, \times \, ED \, }}{BW \, \times \, AT}$$

*C*_*soil*_ is HM concentration in soil (mg/kg). Refer to Table 1 for *BW*, *ED*, *CF*, *SA*, *AF*, *IR*_*ing*_, *ABF*, *AT*, *EF*, and other HM exposure risk parameters.

**Table 1:** Calculation parameters and values used in health-risk assessment to evaluate soil exposure risk with Monte Carlo simulation.

Parameters	Unit	Children	Female	Male	Probabilistic dstribution	References	
Average body weight (BW)	kg	(19.6, 1.96)	(57.59,8.3)	(67.55,8.72)	Lognormal	Men et al.^46^	
Exposure duration (ED)	Year	(0,6)	(0,24)	(0,24)	Uniform	Sun et al.^29^	
Skin area exposed to soil (SA)	cm^2^	50th:2074; 95th:2493	50th:5039; 95th:5938	50th:5039; 95th:5938	Normal	Sun et al.^29^	
Skin adherence factor	mg/(cm^2^d)	0.2(0,3.3)	0.07(0,0.3)	0.07(0,0.3)	Beta-PERT	Men et al.^46^	
Ingestion rate (IR_ing_)	mg/day	50th:100; 95th:200	50th:50; 95th:200	50th:50; 95th:200	Lognormal	Duan^45^	
Average time (AT)	Day	365 × ED (noncarcinogenic)			Point	MEP^47^	
		365 × 70 (carcinogenic)					
Exposure frequency (EF)	Day/year	350 (180,365)	350 (180,365)	350 (180,365)	Triangular	Sun et al.^29^	
Dermal adsorption factor	–	0.001(noncarcinogenic)			Point	USEPA^48^	
(ABF)	kg/mg	0.01(carcinogenic)					
Conversion factor (CF)	–	1 × 10^–6^			Point		

(a1, a2) defines the mean and standard deviation of lognormal distribution; (b1, b2) defines the minimum and maximum values of uniform distribution; 50th and 95th are the values at 50% and 95% of normal distribution, respectively; c1 (c2, c3) defines the most likely, minimum, and maximum values of beta PERT distribution, respectively; and d1 (d2, d3) defines the most likely, minimum, and maximum values of triangular distribution, respectively.

NCR is assessed by the total hazard index (HI), and CR is assessed by the total carcinogenic risk (TCR) of HMs in the soil, calculated as follows^31^:

4 $$TCR = \sum\limits_{i = 1}^{n} {CR_{i} = \sum\limits_{i = 1}^{n} {(ADD_{i} } } \times SF_{i} )$$

5 $$HI \, = \, \sum HQ_{i} \, = \, \sum \frac{{ADD_{i} }}{{RfD_{i} }}$$

where *CR*_*i*_ is the CR of each HM, *SF*_*i*_ is the carcinogenic slope factor of each HM, and its reference is shown in Table 2. If TCR > 10^–4^, the risk is unacceptable; if TCR < 10^–6^, the risk is the opposite. NCR was evaluated by HI. *HQ*_*i*_ is the hazard quotient of each HM, and *RfD*_*i*_ is the corresponding reference value of each HM. When HI < 1, NCR is acceptable; when 1 < HI < 4, NCR is moderate; and when HI > 4, NCR is high.

**Table 2:** Reference dose (*RfD*) and slope factor (*SF*) values of heavy metal(loid)s in soil by different exposure pathways used in health-risk assessment with Monte Carlo simulation.

HM	*RfD* (mg/(kg d))		*SF* ((kg d)/mg)	
	Ingestion	Dermal	Ingestion	Dermal
Zn	0.3^a^	6.0E-02^a^	–	–
Cu	4.0E-02^a^	1.2E-02^a^	–	–
Cr	3.0E-3^a^	6.0E-05^a^	0.5^a^	20^a^
Pb	3.50E-03^a^	5.25E-04^a^	8.5E-03^b^	–
Hg	3.0E-04^a^	2.1E-05^a^	–	–

^a^Sun et al.^29^; ^b^USEPA^48^.

### Hybrid model combining Unmix and HRA

The health risks of HMs from different sources were quantitatively evaluated by combining the Unmix and HRA models. This hybrid model involves four steps:

A) Analyze HM sources based on Unmix.
B) Calculate HM concentration in each sample from each source. The calculation formula is as follows:
  6 $$C_{ij \, }^{k} { = }F_{ij \, }^{k} \times X_{ij}$$

where ${\text{C}}_{\text{ij}}^{\text{k}}$ is the concentration of each HM in each sample from each source, ${\text{F}}_{\text{ij}}^{\text{k}}$ is the estimated contribution rate of the *i* element in the *k*th source in the *j*th sample, and *X*_*ij*_ is the measured concentration (mg/kg) of the *i* element in the *j*th sample.

C) Fit the probability distribution curve of the *i*th element of the *k*th source in all samples.
D) Quantitatively evaluate the human health risks of different HM sources using Monte Carlo simulation. HM health risks from each source are added by the *i*th element of the *k*th source in the *j*th sample. The calculation formula is as follows:
  7 $$ADD_{ij,ing}^{k} \, = \, \frac{{C_{ij}^{k} \, \times \, IR_{ing} \times CF \times \, EF \, \times \, ED \, }}{BW \, \times \, AT}$$
  8 $$ADD_{ij,dermal}^{k} \, = \, \frac{{C_{ij}^{k} \, \times CF \, \times SA \, \times \, AF \, \times \, ABF \, \times \, EF \, \times \, ED \, }}{BW \, \times \, AT}$$

The CR $CR_{ij,n}^{k}$ and NCR $HQ_{ij,n}^{k}$ of HMs from different sources were determined by

9 $$CR_{ij,n}^{k} { = (}ADD_{ij,n}^{k} \times SF_{i} {) }$$

10 $$HI = HQ_{ij,n}^{k} = \frac{{ADD_{ij,n}^{k} }}{{RfD_{i} }}$$

where $CR_{ij,n}^{k}$ is the CR of the *k*th source to the *n*th exposure path in the *j*th sample, and *SFi* is the slope factor for each HM (Table 2). NCR from different sources is determined by the formula where $HQ_{ij,n}^{k}$ is the hazard factor for the *k*th source of the *i*th metal in the *j*th sample on the *n*th exposure path.

### Statistical analysis

Excel 2019 was used for the descriptive statistics. EPA Unmix 6.0 was used for the quantitative analysis of sources. The Monte Carlo risk simulation of HRA was based on Crystal Ball 11.1.2.4. Origin 2021 was used for the risk distribution probability map.

## Results

### Descriptive statistics of soil HMs

Table 3 shows the descriptive statistics for soil HMs in the study area. The mean values of Zn, Cu, Cr, Pb, and Hg in the soil were 55.34, 27.16, 37.31, 79.60, and 0.03, respectively. Compared with the background values of HMs in the soil, except for Zn and Cr, other elements exceeded the standard to varying degrees. Cu was slightly higher than the background value of the soil in Xinjiang, and Pb and Hg were 4.1 and 2.0 times the background value of the soil, respectively. In addition, the maximum content of all elements in the region was higher than the background value of the soil. Compared with the national risk screening value of agricultural land, the average value of all HMs did not exceed the risk threshold, but the values of Cu and Pb at some sites did exceed the risk threshold. In addition, the coefficient of variation of HMs ranged from 0.22 to 0.36, indicating that the soil environment in the study area was strongly affected by human activity. Furthermore, HM concentration in the soil had a high degree of spatial heterogeneity. These results indicate that HM contamination of soil in the region is serious, and the possible health risks need to be emphasized.

**Table 3:** Descriptive statistical results for the heavy metals in the soil.

HM	Range	Mean	CV	Background value of Xinjiang soil	National standard
	(mg·kg^-1^)	(mg·kg^-1^)	(%)	(mg·kg^-1^)	(mg·kg^-1^)
Zn	20.63–132.06	55.34	0.22	68.8	250
Cu	6.38–119.19	27.16	0.36	26.7	100
Cr	9.06–71.25	37.31	0.28	49.3	200
Pb	36.25–129.38	79.60	0.24	19.4	120
Hg	0.01–0.06	0.03	0.28	0.017	2.4

Background values are based on the background values for soil elements in Xinjiang (Zhang et al.^49^). National standard values are based on soil environmental quality standards (GB 15,618–2018).

### Source analysis of HMs in soils

**Figure 2:**
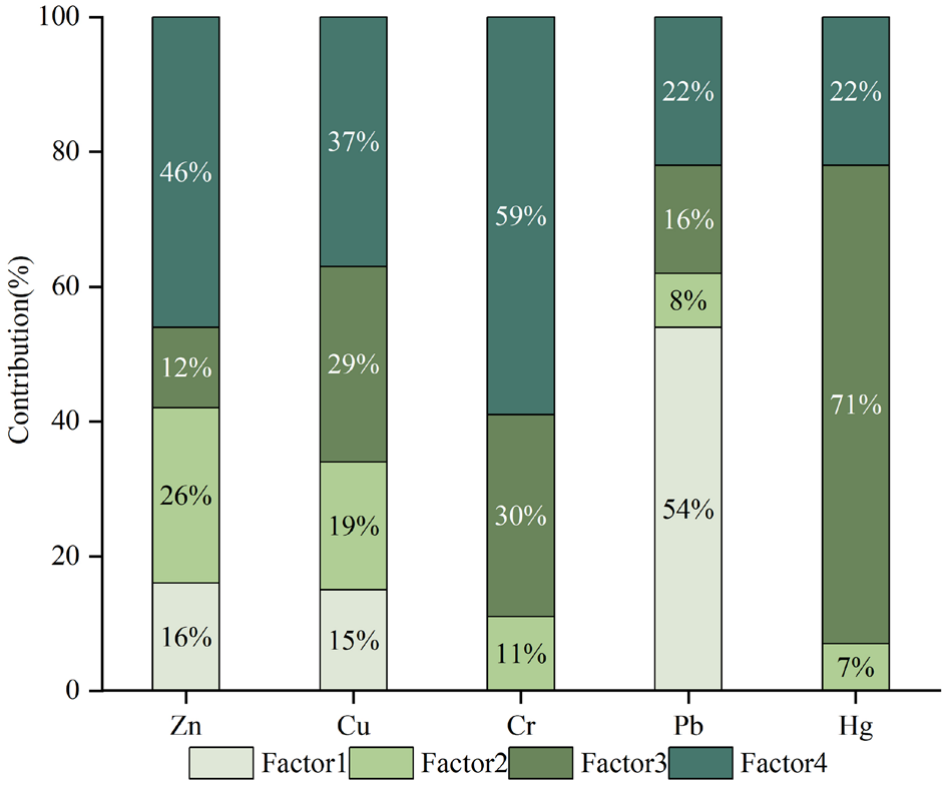
Unmix results. Software: Origin2021. URL: https://www.originlab.com/.

Unmix was used to quantitatively analyze the possible sources of HMs in the soil. When using Unmix, data do not need to be standardized, which could change the pollution source information and affect the accuracy of the results. After eliminating outliers, the data were input into Unmix. The total HMs concentration was set to Total and Norm, and the model was run. At this time, the minimum R^2^ was 0.98, and the minimum signal-to-noise ratio was 2.23, indicating that the quantitative analysis results were reliable. Figure 2 shows the quantitative analysis results.

The main loadings on factor 1 are Pb (55%), Zn (16%), and Cu (15%); Cr and Hg have no loadings on the source. Previous studies have indicated that the main source of Pb in the soil is transportation emissions. The wear of automobile engines and the combustion of lead-containing gasoline will emit Pb^32,33^. Meanwhile, the wear of automobile tires and related galvanized parts will emit Zn and Cu^34^. Roads are dense in the study area, which is an important logistics and transportation hub in Xinjiang. Traffic flow is large, and there is frequent movement of large coal transport vehicles in the Zhundong coalfield. High-frequency traffic activity emits these HMs into the soil; thus, factor 1 represents the traffic source. This is consistent with the results of previous studies^35,36^.

Factor 2 had higher loadings on Zn (26%), Cu (19%), and Cr (11%) and lower loadings on Pb (8%) and Hg (7%). The mean values of Zn and Cr in the study area were lower than the soil background value in Xinjiang. The mean value of Cu was equivalent to the soil background value and was far lower than the national secondary standard. Some studies have investigated how the weathering of soil parent materials and rock components produce Zn and Cu, among others^37^. Therefore, factor 2 is likely to represent natural sources.

Factor 3 had the highest loadings on Hg (71%), Cr (30%), and Cu (29%), followed by Pb (16%); it had a low loading on Zn (12%). Many studies have shown that Hg in the soil is strongly related to coal. As mentioned previously, the large Zhundong coalfield is near the study area. There are also many coal-related industrial and mining enterprises in the study area. Coal dust formed by coal mining and accumulated coal gangue will transfer a large of HMs to the soil. Cr is the result and proof of coal-dust diffusion^38^. In addition, the southern part of the study area has the densest urban agglomeration in Xinjiang. Urban development requires a great deal of electricity. China’s power sources are still dominated by coal combustion, which is an important source of soil Hg. Therefore, factor 3 represents the coal source.

Factor 4 had the highest loadings on Cr (59%), followed by Zn (46%), Cu (37%), Hg (22%), and Pb (22%). Previous studies have shown that industrial emissions of Cr will indirectly enter the soil through waste gas, wastewater, and solid waste. There are many coal processing–related industrial enterprises in the study area^39^, such as coal washing and metal smelting, resulting in increases in Cr in the soil environment. Meanwhile, studies have also shown that industrial production is related to Zn, Cu, and Pb in the soil environment. For example, Cu and Pb can enter the soil from burning fuel. The smelting and electroplating industries will also discharge Cu-containing compounds into the soil^40^. Zn is an excellent anticorrosion material. Galvanized materials are widely used, and their production processes will produce pollution. At the same time, mining, coal combustion, and battery manufacturing also produce Zn. Thus, factor 4 represents the industrial source.

### HRA based on Monte Carlo simulation

Using Oracle Crystal Ball, Monte Carlo simulation was used to evaluate the health-risk probability of soil HM concentration and pollution sources. Previous studies have shown that simulation results are stable after 10,000 simulations^41^; thus, the number of simulations was set to 10,000.

### Concentration oriented HRA

**Figure 3:**
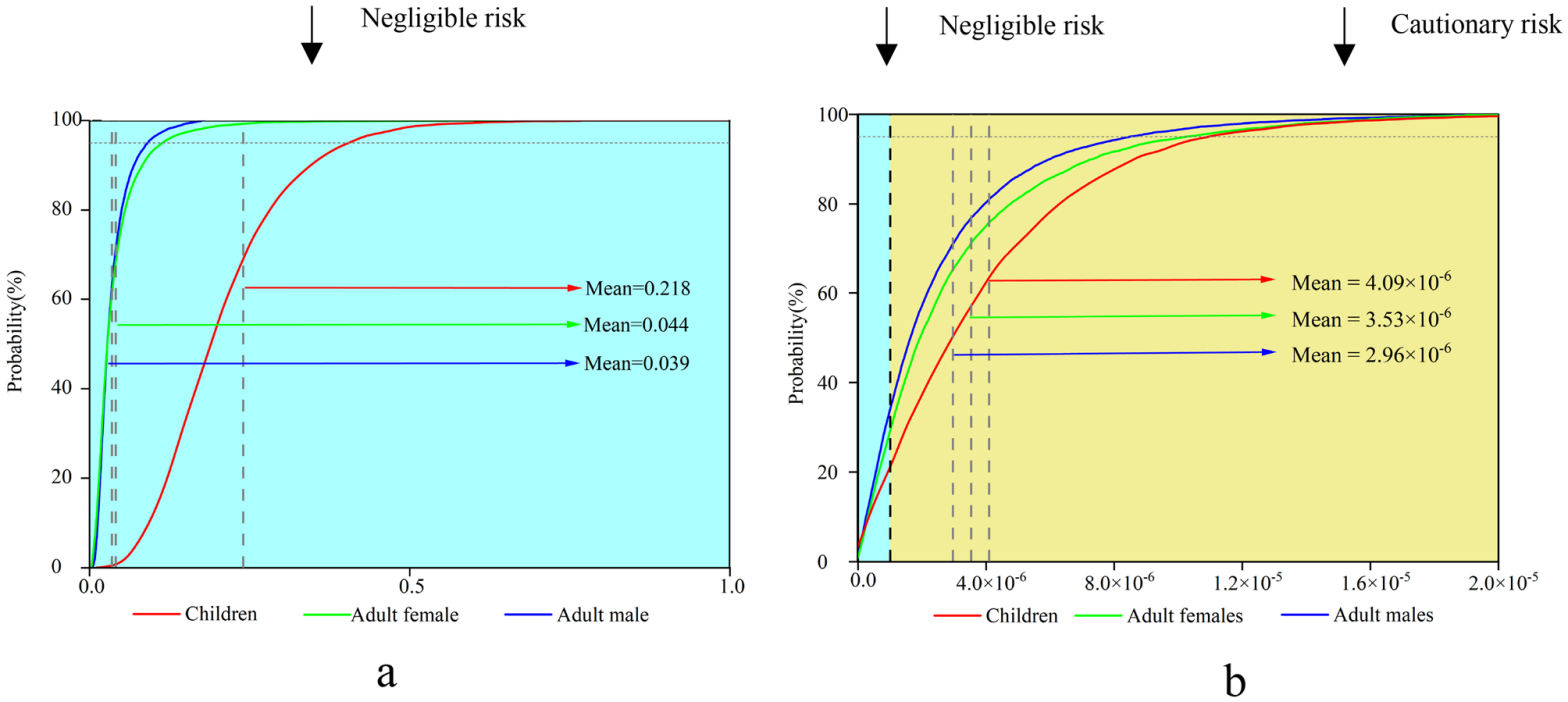
Distribution for TCR and HI: (**a**) total carcinogenic risk (TCR); (**b**) hazard index (HI). Software: Origin2021. URL: https://www.originlab.com/.

The health-risk probability distribution for children, females, and males in the study area was evaluated using Monte Carlo simulations. Figure 3 shows the HI distribution for different populations. The mean HI of the three groups from large to small was children (0.218), females (0.044), and males (0.039). According to the probability distribution, the HIs of children, women, and men were all below the critical value recommended by USEPA (HI = 1) and within the safe range. The HI of all populations in this study area was below the acceptable risk threshold. In summary, there was no significant NCR of HMs in soils for regional populations; it can therefore be ignored.

Figure 3b shows the probability distribution of TCR in soil HMs in the study area. The mean TCR of each group is in descending order of children (4.09 × 10^–6^), females (3.53 × 10^–6^), and males (2.96 × 10^–6^). The TCR values of nearly 77.52% of children, 69.09% of adult women, and 65.63% of adult men in the study area exceeded the critical value of 1 × 10^–6^. This result shows that the CR of soil HMs in the study area is high, which might lead to a higher prevalence of cancer among the population in the region^42^. Therefore, the CR of soil HMs in the study area cannot be ignored and requires attention. Moreover, children were found to have a more serious CR than adults. Children are usually more susceptible to soil pollution than adults. This is consistent with previous research results^43^. On the one hand, children are lighter in weight; on the other hand, it relates to children’s higher oral intake rate and skin adsorption factor. It is necessary, therefore, to keep hands and mouth clean (e.g., avoid sucking fingers), and children should be cleaned in a timely manner after contact with soil^44^. Thus, sensitive groups—namely, children—should be prioritized in source-oriented HRA in the study area.

### Source oriented CR assessment

The concentration-oriented health assessment of HMs in soil can help us intuitively understand pollution levels, but it cannot help decision-makers control the sources of HM pollution in soil^29^. It is necessary, therefore, to carry out the HRA of HMs from different sources. In the source-oriented assessment of health risks, it is necessary to use Monte Carlo simulation to fit the distribution of HM concentration in each source. Table 4 shows the probability distribution types and key parameters of different sources of HMs.

**Table 4:** Fitting types and parameters of the probability distribution of different HM sources.

Elements	Factor 1	Factor 2	Factor 3	Factor 4
Zn	S-T (8.85,1.11,)	S-T (14.39,1.80)	S-T (6.64,0.83)	S-T (25.46,3.18)
Cu	I (2.37,0.38)	I (5.01,0.8)	I (7.65,1.22)	I (9.76,1.55)
Cr	–	I (4.07,0.63)	I (11.11,1.73)	I (21.85,3.40)
Pb	M (38.81,8.91)	M (5.56,1.3)	M (111.29,2.59)	M (15.53,3.57)
Hg	–	I (0.00,0.00)	I (0.02,0.00)	I (0.01,0.00)

(a1, a2) in S-T defines the midpoint and scale of the student distribution, (b1, b2) in I defines the mean and scale of the logical distribution, and (c1, c2) in M defines the most likely value and scale of the maximum extreme value distribution.

### Results for source-oriented CR assessment

**Figure 4:**
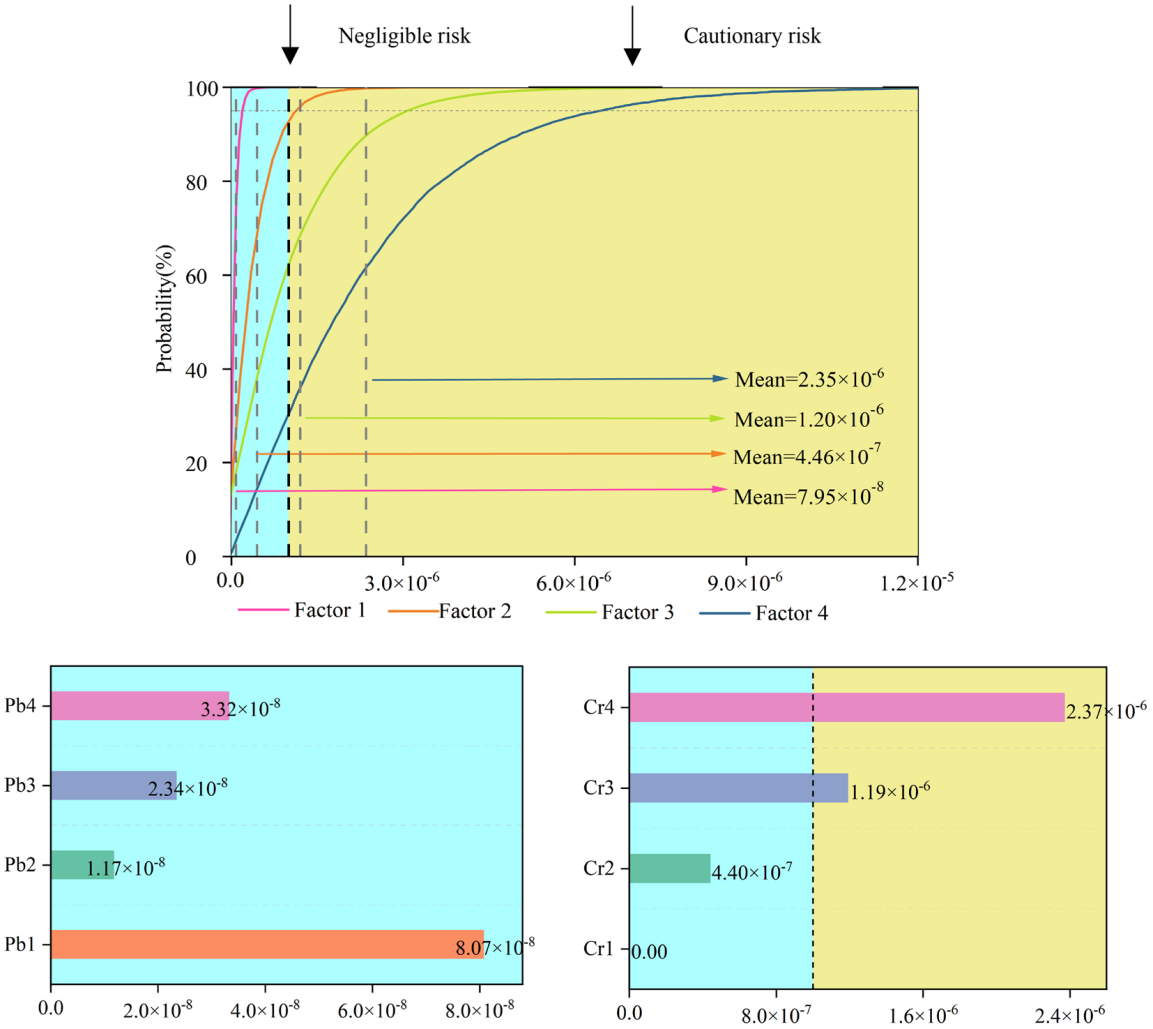
Probability distribution of total carcinogenic risk for children and carcinogenic risk from heavy metals based on each source. Software: Origin2021. URL: https://www.originlab.com/.

Figure 4 shows the probability distribution of CR for children and the CR of HMs from different sources. From large to small, the mean CR values were factor 4 (2.35 × 10^–6^), factor 3 (1.20 × 10^–6^), factor 2 (4.46 × 10^–7^), and factor 1 (7.95 × 10^–8^). The contribution rate of Pb to CR in the four sources was far less than 1.0 × 10^–6^. The industrial source was the main source of CR in the study area, accounting for 57.7% of the total carcinogenic risk contribution rate of children. Its mean value was 2.35 times the acceptable CR threshold (1 × 10^–6^), indicating that CR is serious. The source of the second-highest CR risk was factor 3 coal source, accounting for 29.4% of the CR. The mean value exceeded the acceptable threshold by 1.20 times, and the mean values of factors 1 and 2 were below the acceptable CR threshold. Cr was the main element contributing to CR in the study area, which is related to the lighter weight of children and the higher risk of Cr skin contact carcinogenesis (*SF*). Cr emissions from industrial and coal sources in the study area pose a higher CR to human health in the region. Therefore, in the future, more attention should be paid to industrial activities and coal mining in the region. The planning of residential gathering areas should be as far away from factories as possible. The activities of the mining industry should be further rationally planned to ensure that residents in the region are not affected by the carcinogenic risk of HMs.

### Source-oriented NCR assessment

**Figure 5:**
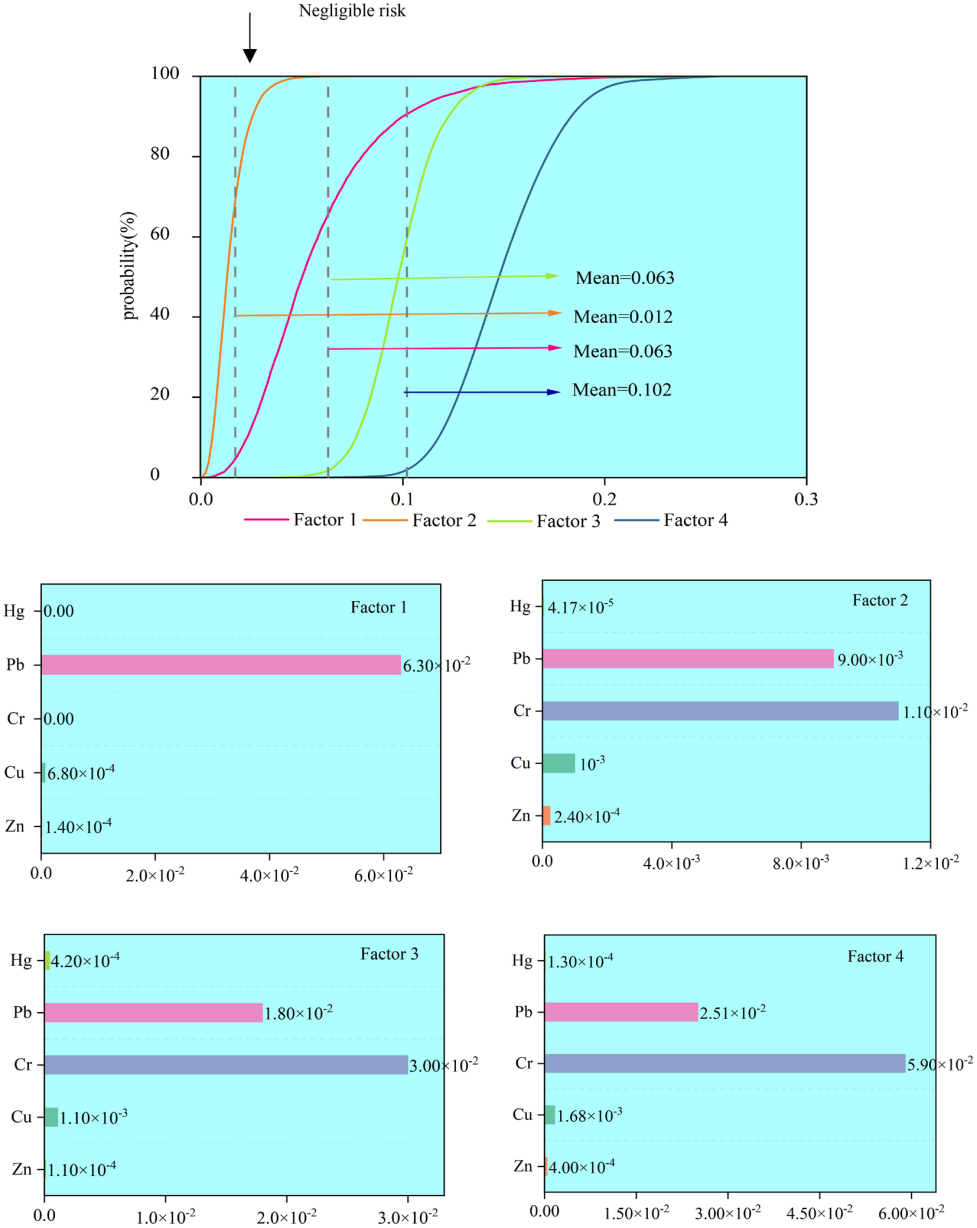
Probability distribution of the total NCR for children based on different sources and the NCR of each HM. Software: Origin2021. URL: https://www.originlab.com/.

Figure 5 shows the probability distribution of NCR for children and the NCR of HMs from different sources. The mean value of the NCR of each source was roughly consistent with the mean value of CR: factor 4 (0.102) > factor 3 = factor 1 (0.063) > factor 2 (0.017). Among them, factor 4 (industrial sources) is more important for HI than other sources, mainly from the contribution of Pb and Cr elements. The HI of all sources was less than 1, which is within the acceptable range, indicating that the potential NCR for children in the study area can be ignored.

## Discussion

In this study, 170 soil samples were systematically collected from Fukang, Jimsar, Qitai and other areas on the northern slope of the Tianshan Mountains in Xinjiang, China. A new receptor model Unmix model was used to analyze the source of soil heavy metals. The R^2^ of the source analysis results reached more than 0.98, and the minimum signal-to-noise ratio S/N reached 2.23, which proved that the source analysis results were reliable. In future studies, other receptor models such as PMF and APCS-MLR can be combined to further improve the interpretability and reliability of the source analysis results. In addition, the human health risk assessment model is considered to be an effective method to determine the degree of harm of soil heavy metals to human body. However, the traditional human health risk assessment model is subject to fixed parameters and the use of a limited number of heavy metal content data. It is difficult to avoid errors in human health risk assessment^23^. In this study, Monte Carlo simulation was used in combination with human risk assessment model to eliminate this error to a certain extent, and the sources of Unmix model analysis were used for Monte Carlo simulation of human health risk. The health risks of heavy metals from various sources to sensitive people and the contribution rate of various sources to human health risks were obtained, and the primary control pollution sources were determined. However, at the same time, this study only evaluates the human health risks of five kinds of soil heavy metals, so the human health risk assessment in the study area is still incomplete and limited. In future research, the effects of As and Cd on human health risks should be taken into account. Through the soil heavy metal assessment pollution assessment model and the spatial distribution of soil heavy metals, a more comprehensive understanding of the pollution degree and spatial distribution of heavy metal contaminated soil can be obtained to avoid the health threat of soil heavy metals to the population.

## Conclusion

The study area in the present research included Fukang, Jimsar, Qitai, and Midong in the economic belt of the northern slope of Tianshan Mountains in Xinjiang, China. The soil environment in this area is seriously polluted by HMs. Soil samples were taken from the area to conduct HRA on the sources of soil HMs. The results showed the following. (1) The mean values of Zn and Cr lover the background value of Xinjiang soil, but the maximum value exceeded the background value of Xinjiang soil. The mean values of Pb and Hg were 4.1 and 2.0 times of the background value of Xinjiang soil, which were lower than the national standard. (2) Unmix analysis showed that the main sources of HMs were traffic, natural, coal, and industrial sources. Cr (59%), Zn (46%) and Cu (37%) were mainly from industrial sources, Hg (71%) was mainly from coal sources, and Pb (54%) was mainly from traffic sources. (3) The concentration-oriented Monte Carlo HRA showed that the NCR of children was 0.218, that adult females was 0.044, and that of adult was 0.039, all within the acceptable range, while CR was at a high risk. The mean value of children was 4.06 × 10^–6^, that of adult females was 3.53 × 10^–6^, and that of adult males was 2.96 × 10^–6^. Children have higher NCR and CR than adults because of their lighter weight and higher oral intake rate. Children are therefore the most sensitive population in the study area. (4) Source-oriented HRA based on Unmix and Monte Carlo quantitatively analyzed the relationship between HMs, pollution sources, and health risks for the sensitive population. The average CR of industrial sources exceeded the acceptable threshold by 2.35 times, the average CR of coal sources exceeded the threshold by 1.20 times, and the average value of natural sources and traffic sources was below the acceptable threshold. Among them, Cr was the main element contributing to CR. Based on the findings, industrial sources should be the main HM source factors that are preferentially controlled in the region. Furthermore, Cr emitted from coal sources also requires attention.

## Data Availability

The data that support the findings of this study are available on request from the corresponding author, Abdugheni Abliz, upon reasonable request.
